# Supplementary material for: Modeling glioblastoma heterogeneity as a dynamic network of cell states
Source: Mol Syst Biol. 2021 Sep 16;17(9):e10105. doi: 10.15252/msb.202010105 (PMC8444284; doi:10.15252/msb.202010105)
Supplement: Supplementary file 6 — Source Data for Figure 5 [file MSB-17-e10105-s004.zip › Figure5A_sourcedata/GSEA_3017/hallmarks_stateD.GseaPreranked.1621934542601/HALLMARK_FATTY_ACID_METABOLISM.html]

Details for gene set HALLMARK\_FATTY\_ACID\_METABOLISM[GSEA]

|  || Dataset | state23017 |
| Phenotype | NoPhenotypeAvailable |
| Upregulated in class | na\_pos |
| GeneSet | HALLMARK\_FATTY\_ACID\_METABOLISM |
| Enrichment Score (ES) | 0.23512618 |
| Normalized Enrichment Score (NES) | 1.2749566 |
| Nominal p-value | 0.15428571 |
| FDR q-value | 0.32709435 |
| FWER p-Value | 0.49 |
Table: GSEA Results Summary

  

Fig 1: Enrichment plot: HALLMARK\_FATTY\_ACID\_METABOLISM      
 Profile of the Running ES Score & Positions of GeneSet Members on the Rank Ordered List

  

| PROBE | GENE SYMBOL | GENE\_TITLE | RANK IN GENE LIST | RANK METRIC SCORE | RUNNING ES | CORE ENRICHMENT || 1 | IDI1 |  |  | 34 | 0.382 | 0.0354 | Yes |
| 2 | SMS |  |  | 45 | 0.358 | 0.0854 | Yes |
| 3 | MIF |  |  | 48 | 0.351 | 0.1404 | Yes |
| 4 | LGALS1 |  |  | 83 | 0.308 | 0.1639 | Yes |
| 5 | LDHA |  |  | 92 | 0.297 | 0.2055 | Yes |
| 6 | SERINC1 |  |  | 181 | -0.252 | 0.1785 | Yes |
| 7 | SUCLG1 |  |  | 200 | -0.253 | 0.2055 | Yes |
| 8 | EPHX1 |  |  | 216 | -0.255 | 0.2351 | Yes |
| 9 | FH |  |  | 408 | -0.274 | 0.1326 | No |
| 10 | PRDX6 |  |  | 475 | -0.282 | 0.1274 | No |
| 11 | ALDH9A1 |  |  | 483 | -0.283 | 0.1676 | No |
| 12 | HSDL2 |  |  | 611 | -0.303 | 0.1188 | No |
| 13 | NCAPH2 |  |  | 616 | -0.303 | 0.1646 | No |
| 14 | ACADM |  |  | 636 | -0.308 | 0.1996 | No |
| 15 | HADH |  |  | 754 | -0.333 | 0.1633 | No |
| 16 | GLUL |  |  | 920 | -0.382 | 0.0980 | No |
| 17 | SDHA |  |  | 951 | -0.396 | 0.1387 | No |
| 18 | CBR1 |  |  | 966 | -0.400 | 0.1924 | No |
| 19 | ALDH3A2 |  |  | 1123 | -0.489 | 0.1513 | No |
Table: GSEA details [plain text format]

  

Fig 2: HALLMARK\_FATTY\_ACID\_METABOLISM: Random ES distribution      
 Gene set null distribution of ES for **HALLMARK\_FATTY\_ACID\_METABOLISM**
